# *Christensenella massiliensis* reduces kynurenine levels and alleviates obesity and related metabolic disorders in model mice

**DOI:** 10.1080/19490976.2026.2701382

**Published:** 2026-07-11

**Authors:** MengXuan Du, Wenzhao Wang, Min-Zhi Jiang, Xin-Wei Sun, Lei Sun, Chang Liu, Shuang-Jiang Liu

**Affiliations:** a State Key Laboratory of Microbial Technology, Shandong University, Qingdao, China; b State Key Laboratory of Microbial Diversity and Innovative Application, Institute of Microbiology, Chinese Academy of Sciences, Beijing, China; c Shandong Provincial Key Laboratory of Animal Cell and Developmental Biology, School of Life Sciences, Shandong University, Qingdao, China

**Keywords:** *Christensenella massiliensis*, obesity, metabolic disorder, kynurenine

## Abstract

Next-generation probiotics derived from gut commensals show promise for metabolic disease intervention, yet effective anti-obesity strains remain limited. Here, we demonstrate that oral administration of *Christensenella massiliensis* markedly alleviates obesity and metabolic dysfunction in high-fat diet-induced obese mice. Treatment reduced food intake, improved glucose tolerance and insulin sensitivity, lowered blood glucose and lipid levels, and attenuated hepatic steatosis and adipose accumulation. *C. massiliensis* increased the levels of plasma GLP-1 and ileal GLP-1 receptor expression while decreasing ghrelin level, suggesting modulation of gut hormone regulation. *C. massiliensis* also suppressed systemic and colonic inflammation, accompanied by upregulation of metabolic homeostasis-related genes (*ppara*, *pparg*, *ucp2*). Targeted and quantitative metabolomics identified altered gut metabolic profiles, particularly reduced kynurenine levels. *In vitro* assays further showed that *C. massiliensis* converted kynurenine into kynurenic acid, and its lysate reversed kynurenine-induced lipid accumulation, inflammation, and PPARγ suppression in hepatocytes, providing mechanistic support for the observed *in vivo* metabolic benefits. These findings support *C. massiliensis* as a promising next-generation probiotic for obesity management.

## Introduction

Current studies reveal that gut microbiota plays an important role in host obesity and metabolic disorders, including type 2 diabetes, cardio-metabolic disease, and non-alcoholic fatty liver disease, posing significant threats to human health.^1‐5^ Current weight management strategies primarily include dietary modifications, physical activity, pharmacological interventions, and bariatric surgery.^6‐8^ While dietary and exercise interventions typically require prolonged periods to achieve substantial weight reduction, surgical approaches carry significant risks and potential complications. Emerging evidence suggests that probiotic supplementation represents a promising alternative for weight management. In recent years, the development of specialized probiotic formulations for weight loss has gained increasing attention.^9^ The utilization of commensal gut microbiota for weight regulation offers several advantages, including reduced adverse effects compared to pharmacological interventions and the absence of dependency risks associated with certain weight-loss medications.

Among weight-management probiotics, members of the *Christensenellaceae* family have attracted particular attention due to their significant negative correlation with body mass index (BMI) in cohort-based studies.^10‐13^ Recent mechanistic studies have demonstrated that *Christensenella minuta* enhances host metabolic health by producing a novel class of secondary bile acids, 3-O-acyl bile acids, which function as intestinal farnesoid X receptor (FXR) antagonists.^14^ Considering the diverse species resources and their diverse metabolic ability,^15^ the members of *Christensenellaceae* family may interact with host via different molecules or pathways. For example, *Luoshenia tenuis*, a recently described genus of *Christensenellaceae* family, regulates host energy homeostasis by modulating circulating levels of peptide YY and ghrelin, thereby reducing food intake and adiposity.^16^ Interestingly, the study by Liu et al. systematically evaluated the 3-O-acyl bile acid biosynthetic capacity of 88 core gut microbial strains and disclosed that another member of the *Christensenella* genus, *Christensenella massiliensis*, lacks the ability to synthesize this class of bile acids. The *C. massiliensis* may exert its physiological effects through alternative mechanisms of host interaction, which warrant further investigation.

Previous studies demonstrated that imbalances in amino acid metabolism contribute to various metabolic diseases.^17‐24^ For instance, elevated kynurenine levels, a key metabolite in the tryptophan-kynurenine pathway, contribute to obesity through mechanisms involving inflammatory cytokine modulation and disruption of energy balance, leading to increased fat accumulation and insulin resistance.^25^ ^,^ ^26^ Kynurenine metabolites, such as kynurenic acid, have been shown to impact metabolic pathways by influencing adiposity regulation and fat storage.^27^ ^,^ ^28^ Another example is that branched-chain amino acids (BCAAs), which are essential amino acids for humans, have been linked to metabolic diseases, as elevated BCAA levels contribute to insulin resistance and atherosclerosis. Recently, modulating gut microbiota to regulate amino acid metabolism, as exemplified by *Parabacteroides merdae* in preventing atherosclerosis through BCAA catabolism, has been proved to be a promising strategy for treating metabolic diseases.^29^ This evidence suggests that targeting host-unfavorable amino acids and their metabolites, such as kynurenine, through microbiota-based interventions could offer a feasible approach for alleviating obesity and associated metabolic disorders. Moreover, substantial evidence indicates that obesity not only impairs host glucolipid metabolism but also triggers chronic low-grade inflammation, which further exacerbates insulin resistance and increases the risk of cardiovascular and cerebrovascular diseases.^30^ ^,^ ^31^ Therefore, improving metabolic health by regulating amino acid metabolism and reducing inflammation has become a key focus of current research. In recent years, probiotics and NGPs have garnered significant attention for their potential to modulate gut microbiota, improve metabolic health, and alleviate inflammation.^32‐34^ The development of NGPs capable of effectively clearing excess amino acids, reducing inflammation, and restoring energy metabolism balance has emerged as a promising strategy for addressing obesity and metabolic disorders.

In this study, we demonstrated that administration of *C. massiliensis* significantly reduced body weight gain and effectively improved metabolic disorders in diet-induced obesity (DIO) mice. Analysis of gut metabolome revealed a significant reduction in obesity-associated amino acid derivatives, particularly kynurenine, following treatment. *In vitro* experiments confirmed that *C. massiliensis* could metabolize kynurenine into kynurenic acid. Further gene expression profiling of DIO mice showed upregulation of energy metabolism-related genes in the liver and colon following *C. massiliensis* treatment. These results suggested that the catabolism of kynurenine by *C. massiliensis* played a key role in its beneficial effects on obesity and metabolic disorders.

## Materials and methods

### Preparation of bacterial cultures

*C. massiliensis* SJ-1 (CGMCC No. 29796) was grown in a modified mGAM medium at 37°C in an anaerobic environment for 2 d.^35^ The cells were harvested by centrifugation at 6000 ×* g* for 10 min at 4°C. The cells were resuspended in sterile anaerobic PBS (containing 1 g/L L-cysteine) to a final concentration of 1 × 10^9^ CFU per 200 μL.

### Animals and diets

Male C57BL/6J mice were obtained from GemPharmatech Co., Ltd. (Jiangsu, China). They were kept in a biosafety shelter level 2 (BSL-2) laboratory with specific pathogen‑free (SPF) conditions, including a 12-h light/dark cycle, a temperature range of 20°C–22°C, and 45% ± 5% humidity. The mice had free access to food and water, and were acclimated for one week prior to the experiment.

For the *C. massiliensis* assay on high fat diet (HFD) feeding mice, 6-week-old C57BL/6J male mice fed with HFD (D12492i, 60% calories from fat, Research Diets, USA) for 10 weeks were sorted into 2 groups (*n* = 5) randomly for 1 additional week adaption. Mice in DIO_CMS group were gavaged daily for 5 weeks with 1 × 10^9^ CFU of living *C. massiliensis* suspended in 200 μL of sterile anaerobic PBS, while DIO_CK group was given the same volume of sterile anaerobic PBS. All mice experiments in this study were approved by the ethics committee of Institute of Microbiology, Chinese Academy of Sciences (IMCAS). The protocols were approved by the Committee on the Ethics of Animal Experiments of IMCAS (permit APIMCAS2020091). The experiments were conducted according to the National Institutes of Health Guidelines for the Care and Use of Laboratory Animals (NIH publications No. 8023, revised 1978).

### Oral glucose tolerance test (OGTT) and insulin tolerance test (ITT)

The OGTT was performed by gavage of glucose solution (2 g/kg) after 12 h fasting. The ITT was performed by injecting insulin (0.6 U/kg) intraperitoneally after 6 h fasting. The level of blood glucose was measured by tail vein blood sampling using a glucose meter (Accu Check, Roche Diagnostics GmbH) at 0, 15, 30, 60, and 150 min after oral glucose load or insulin injection. The area of the curve (AOC) of OGTT and ITT was calculated as recommended by Virtue and Vidal-Puig.^36^

### PCR amplification and sequencing analysis of feces

DNA for amplicon sequencing of gut microbiota was extracted from feces. Each mouse was kept in an empty compartmentalized storage box for 30 min, and then collected fresh fecal samples into sterile tubes. Tubes were stored at −80°C until use. The DNA extraction and purification by using DNeasy PowerSoil Kit (Qiagen, Germany). The V3–V4 region of 16S rRNA was amplified using the primers F341 (CCTACGGGAGGCAGCAG) and R806 (GGACTACHVGGGTWTCTAAT) by PCR and sequenced on an Illumina HiSeq 2500 platform by Megagene (Guangzhou, China). Raw reads were processed using Usearch (v.11), and taxonomic assignment was performed with the SILVA (v.138) reference database. A representative sequence of each ASV (amplicon sequence variants) was assigned by using the data base from the sequencing company. Principal coordinate analysis (PCoA) was performed based on Bray–Curtis distance matrices, and permutational multivariate analysis of variance (PERMANOVA) was conducted using the R package vegan (v2.6‑10). PCoA plots were generated using the ggplot2 package (v3.5.2).

### Real-time qPCR analysis

Total RNA was isolated and purified from ileum, colon and liver tissues using the TRIzol reagent protocol (Vazyme, China). RNA concentration was measured with a Qubit 4.0 (Invitrogen, Q33226). The cDNA was performed using the HiScript III RT SuperMix reverse transcription kit (Vazyme, China). Gene expression levels were assessed using SYBR Green I (Vazyme, China), with amplification of the target gene carried out using the primers (Table S1). Relative mRNA expression levels, normalized to the GAPDH internal control, were calculated using the comparative threshold cycle (Ct) method,^37^ experiments were performed in triplicate. The qPCR mixture consisted of 10 μL SYBR green polymerase, 1 μL cDNA from ileum tissue, 0.4 μL of each primer, and 8.2 μL RNase- and DNase-free water. The qPCR conditions were as follows: 3 min at 95 °C, followed by 40 cycles of 3 s at 95°C and 30 s at 60°C.

To quantitatively determine the absolute abundance of *C. massiliensis* cells in mouse feces, we designed specific primer sequences (Table S1) and amplified a *C. massiliensis*-specific DNA fragment from metagenomic DNA extracted from fecal samples. The amplified fragment was ligated into the pGM-T vector (TIANGEM, Beijing, China) to generate plasmid p-CMS, which was subsequently used to construct a standard curve from 1 × 10^1^ to 1 × 10^8^ copies of the *C. massiliensis* marker gene. qPCR was performed in a 20 μL reaction mixture containing 50 ng of template DNA and SYBR qPCR Master Mix (Vazyme, China) using a LightCycler 96 system (Roche, Switzerland). The copy number of *C. massiliensis* in each fecal sample was normalized to the annotated 16S rRNA copy number of *C. massiliensis* in the genome, and the results were expressed as the number of cells/mg of metagenomic DNA of fecal samples.

### ELISA, TCHO, and TG assay and histological analysis

The glucagon, ghrelin, GLP-1, and SOD level in ileum, stomach and plasma tissues were determined using commercial ELISA kits, including Mouse Glucagon ELISA Kit, Mouse Ghrelin ELISA Kit, Mouse GLP-1 ELISA Kit, Mouse SOD ELISA Kit, Mouse PPARγ ELISA Kit, Mouse TNF-α ELISA Kit, Mouse IL-6 ELISA Kit, and Mouse IL-1β ELISA Kit (Boshen, Jiangsu, China), following the manufacturer's instruction. The levels of plasma TCHO, triglycerides (TGs), alanine aminotransferase (ALT), and aspartate aminotransferase (AST) were analyzed using commercial kits (Jian-Cheng, Jiangsu, China) following the manufacturer's instructions. The preparation and examination of liver tissue slices stained with hematoxylin and eosin (H&E) and oil red O were performed as described by Qiao et al.^38^, while the hepatocyte ballooning was scored as described by Fujii et al.^39^

### AML12 cells and lipid accumulation induction

The mouse hepatocyte cell line AML12 was purchased from Wuhan Procell Life Science & Technology Co., Ltd. (China). Cells were grown at 37 °C in a 5% CO₂ atmosphere using AML12 Cell Complete Medium (Wuhan Procell Life Science & Technology Co., Ltd., China). Cells were seeded at a density of 1 × 10^5^ cells/mL into 12-well plates and cultured for 24 h. Lipid accumulation was induced using Sodium palmitate/Sodium oleate high-fat cell additive (Dalian Meilun Biotechnology Co., Ltd., China), with final concentrations of 0.25 mM palmitic acid (PA) and 0.5 mM oleic acid (OA). After 24 h of induction, cells were treated for an additional 24 h with the following drugs: 0.1% DMSO, vehicle control, 100 μM kynurenine, 100 μM kynurenine + 10 μg/mL *C. massiliensis* lysate, 10 μg/mL *C. massiliensis* lysate, or 10 μM kynurenic acid.

### Targeted metabolomics of cecal samples

Metabolomic analysis of cecal contents (*n* = 5 biologically independent samples per group) was performed by Metabo-Profile Biotechnology Co., Ltd. (China) using the commercial T500 kit, which covers metabolites including amino acids, free fatty acids, and bile acids. Components exhibiting significant differences were identified based on a Variable Importance in Projection (VIP) score > 1, *p*-value < 0.05, and a logarithmic fold change (Log2 FC) > |1|. Spearman (|r|> 0.8, FDR < 0.05) correlation analysis between the gut metabolome and gut microbiota was conducted using the R package psych (v 2.1.6).

### Growth curve

Carbon-free M9 medium (containing Na_2_HPO_4_ 6.8 g/L, KH_2_PO_4_ 3 g/L, NaCl 0.5 g/L, NH_4_Cl 1 g/L, MgSO_4_ 0.24 g/L, CaCl_2_ 0.01 g/L, pH = 7.2) was used for the sole carbon source experiment. The *C. massiliensis* was cultured in YCFA medium at 37°C under anaerobic conditions for 24 h. The growing cells were harvested by centrifugation at 6000 rpm for 5 min at room temperature, and the supernatant was discarded. The cell pellet was washed twice with carbon-free M9 medium and resuspended in the same medium to adjust the optical density (OD_600_) to 0.5. The adjusted cell suspension was inoculated into fresh carbon-free M9 medium at a 1% (v/v) ratio. For the experimental group, kynurenine was added at a concentration of 25 mM. Each sample was prepared in six replicates, and the OD_600_ was measured every 30 min using a microplate reader (SPECTROstar Omega, Germany).

### Kynurenine, kynurenic acid, and cinnabarinic acid measurement

The *C. massiliensis* was cultured in YCFA medium at 37°C under anaerobic conditions for 24 h. The cells were harvested by centrifugation at 6000 rpm for 5 min at 4 °C and washed twice with PBS buffer. The washed cells were resuspended in YCFA medium and adjusted to an OD_600_ of 1.0. The cell suspension was divided into two parts: (1) 1% (v/v) was inoculated into 5 mL of YCFA medium, and (2) the cell concentration was adjusted to an OD_600_ of 0.5 using YCFA medium, followed by the addition of kynurenine to a final concentration of 1 μM. The control group consisted of YCFA medium containing 1 μM kynurenine without microbial inoculation. All samples were incubated at 37°C under anaerobic conditions for 72 h, with four replicates prepared for each group. Then, the bacterial cells were removed by centrifugation at 12,000 rpm for 10 min. A 3-fold volume of acetonitrile was added to the supernatant, followed by thorough vortexing and centrifugation at 20,000 rpm for 10 min. The solution was then filtered through a 0.22 μm membrane. The medium without bacterial inoculation served as the control, and four replicates were set up for each group. Kynurenine, kynurenic acid, and cinnabarinic acid were used as reference standards for LC-MS/MS analysis. The analysis was performed using a Waters Xevo G3 QT of LC/MS system equipped with an Electrospray ionization (ESI) source. For chromatography separation, a ACQUITY UPLC BEH C8 column (2.1 mm × 100 mm, internal diameter 1.7 μm; Waters) was used at 30 °C with a flow rate of 0.1 mL min-1 for LC separation. The injection volume was 5 μL. The solvent of the mobile phase was 0.1% formic acid in water (A) and acetonitrile (B). Total elution program was 15 min. Gradient began with 5% mobile phase B, changed to 70% B over 10 min, maintained for 1 min and then decreased to 5% over 0.1 min prior to re-stabilization over 4.9 min before the next injection. The gradient program, 95:5 V/V at 0.0 min, 70:30 V/V at 10.0 min, 70:30 V/V at 11.0 min, 95:5 V/V at 11.1 min, and 95:5 V/V at 15.0 min. All MS experiments were detected in the positive ionization mode. For Q-TOF/MS conditions, fragmentor and capillary voltages were kept at 130 and 3500 V, respectively. Nitrogen was supplied as the nebulizing and drying gas. Temperature of the drying gas was set at 300 °C. The flow rate of the drying gas and the pressure of the nebulizer were 11 L/min and 45 psi, respectively. Full-scan spectra were acquired over a scan range of m/z 80–2000 at 1.03 spectra s^−1^.

### Statistical analysis

All data are expressed as the mean ± SEM. Statistical analysis was performed using GraphPad Prism v10. for determination of the proper statistical analysis methods, the normality of the probability distribution was assessed using the Shapiro–Wilk test. For comparisons between two groups, a two-tailed unpaired *t*-test was applied if the data followed a normal distribution. Alternatively, the Mann–Whitney U test was used for non-normally distributed data. For comparisons among multiple groups, one-way ANOVA was used when data followed a normal distribution; otherwise, the Kruskal–Wallis test was applied.

## Results

### *C. massiliensis* alleviated obesity and related metabolic disorders in DIO mice

**Figure 1.**
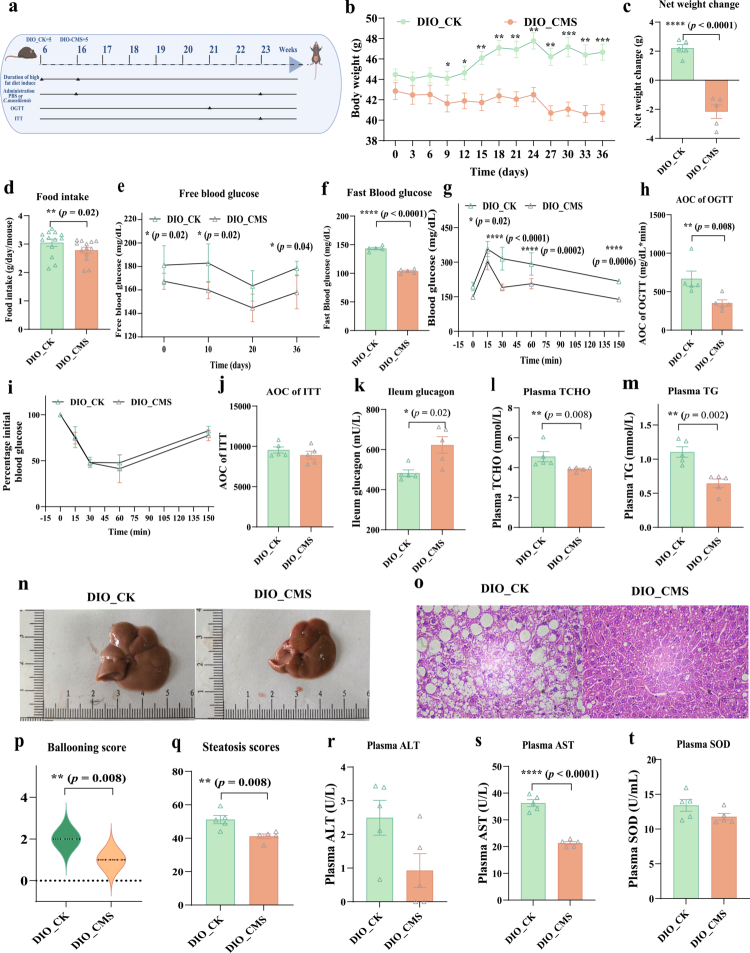
The effects of *C. massiliensis* on body weight and related hormonal level in DIO mice. Flowchart of animal trials (a), the changes of body weight during 5-week treatment (b), the net weight change of mice at the end point (c), average food intake (d), free blood glucose (e), fasting blood glucose of mice at end point (f), plasma glucose profile measured during an OGTT and ITT (g, i), the area of the curve (AOC) of OGTT and ITT (h, j), the level of ileum glucagon (k), the level of plasma TCHO and TG (l, m), hepatic image (n), images of liver after H&E staining (o), the ballooning and steatosis score of liver (p, q), the level of plasma ALT (r), the level of plasma AST (s),the level of plasma SOD (t). DIO_CK: DIO mice that were daily gavaged with 200 μl of PBS (*n* = 5 biologically independent samples); DIO_CMS: DIO mice that were daily gavaged with 10^9^ cells of *C. massiliensis* suspended in 200 μl of PBS (*n* = 5 biologically independent samples). The normality of the probability distribution was assessed using the Shapiro–Wilk test. For comparisons between two groups, a two-tailed unpaired *t*-test was applied if the data followed a normal distribution. Alternatively, the Mann–Whitney U test was used for non-normally distributed data. Data are shown as mean ± SEM. *p<0.05, **p<0.01, ***p<0.001, ****p<0.0001.

To study the effect of *C. massiliensis* on obesity and related metabolic disorders, we conducted an animal gavage experiment with DIO mice for 5 weeks as illustrated in Figure 1a. Treatment with *C. massiliensis* significantly reduced body weight gain in DIO mice, with statistical significance observed from day 15 onward compared to the control group (Figure 1b and c). *C. massiliensis*-treated mice exhibited a significant reduction in food intake (Figure 1d). We next assessed a series of metabolic features of treated and untreated mice before and after end-point sacrifice. The administration of *C. massiliensis* significantly decreased both free blood glucose and fasting blood glucose levels compared to control groups (Figure 1e and f). The glucose tolerance was also significantly improved following treatment, as evidenced by a faster return to baseline glucose levels after the initial peak at 15 min and a reduced area of the curve (AOC) during the oral glucose tolerance test (OGTT, Figure 1g and h). In addition, *C. massiliensis* administration slightly reduced insulin tolerance (ITT), suggesting a potential improvement in insulin sensitivity (Figure 1i and j). Glucagon levels were markedly increased following *C. massiliensis* treatment (Figure 1k). *C. massiliensis* exhibited notable antihyperlipidemic activity, as indicated by significant reductions in plasma total cholesterol (TCHO) and triglyceride (TG) levels (Figure 1l and m). Furthermore, oral administration of *C. massiliensis* resulted in a significant reduction in liver size compared to controls, accompanied by a visible decrease in hepatic lipid accumulation on the liver surface (Figure 1n). H&E staining of liver tissue paraffin sections revealed that hepatic steatosis and ballooning in DIO mice were significantly improved by *C. massiliensis* intervention (Figure 1o). This beneficial effect was corroborated by a significant decrease in ballooning and steatosis scores (Figure 1p and q). In addition, *C. massiliensis* significantly alleviated liver injury in DIO mice, as evidenced by a decrease in plasma alanine aminotransferase (ALT), aspartate aminotransferase (AST), and plasma superoxide dismutase (SOD) levels (Figure 1r–t). These results highlighted the therapeutic potential of *C. massiliensis* against hyperlipidemia and related metabolic disorders. Collectively, these findings demonstrate that *C. massiliensis* exerts beneficial effects on body weight regulation, glycemic control, lipid metabolism, and liver function, highlighting its therapeutic potential for combating obesity-associated metabolic dysfunction and fatty liver lesions in DIO mice.

### *C. massiliensis* improved host metabolism through multi-target regulation

**Figure 2.**
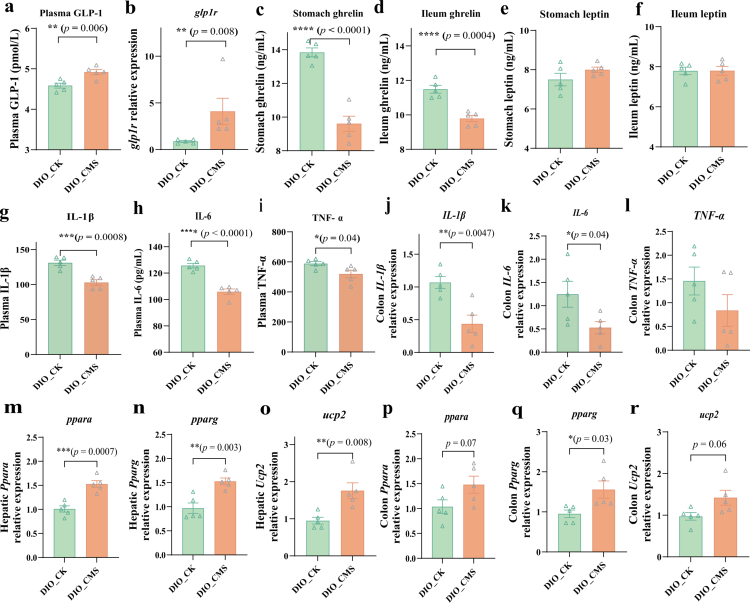
Comprehensive effects of *C. massiliensis* on lipid metabolism, enteroendocrine signaling, hepatic function, inflammatory response, and metabolic homeostasis in DIO mice. The level of plasma GLP-1 (a), and the relative expression of *glp1r* gene in ileum (b), the level of ghrelin in stomach and ileal (c, d), the levels of leptin in stomach and ileum (e, f), the level of IL-1β, IL-6 and TNF-α in plasma (g–i), the relative expression level of *IL-1β*, *IL-6* and *TNF-α* in the colon tissue (j–l). The relative expression level of *ppara*, *pparg*, and *ucp2* in the hepatic tissue (m–o), the relative expression level of *ppara*, *pparg*, and *ucp2* in the colonic tissue (p–r) in DIO_CK and DIO_CMS group (*n* = 5 biologically independent samples for each). The normality of the probability distribution was assessed using the Shapiro–Wilk test. For comparisons between two groups, a two-tailed unpaired *t*-test was applied if the data followed a normal distribution. Alternatively, the Mann–Whitney U test was used for non-normally distributed data. Data are shown as mean ± SEM.

We next examined whether *C. massiliensis* administration affected molecular markers involved in the regulation of host food intake, including ghrelin, leptin, glucagon-like peptide-1 (GLP-1), and the GLP-1 receptor (GLP-1R). *C. massiliensis* treatment significantly increased plasma GLP-1 levels and upregulated GLP-1R expression in the ileum (Figure 2a and b). In contrast, ghrelin levels were markedly reduced in both the stomach and ileum (Figure 2c and d), while leptin levels remained unchanged (Figure 2e and f). These coordinated hormonal changes are known to delay gastric emptying,^40^ ^,^ ^41^ and are consistent with the observed reduction in food intake (Figure 1d).

Furthermore, previous studies have identified metabolic inflammation as a critical driver of obesity, insulin resistance, and fatty liver.^42^ ^,^ ^43^ To evaluate the anti-inflammatory effects of *C. massiliensis*, we quantified representative cytokines including IL-1β, IL-6, and TNF-α in both plasma and colonic tissues of DIO mice (Figure 2g–l). The results showed that all three inflammatory markers were significantly downregulated following *C. massiliensis* treatment, confirming its efficacy in reducing host inflammation. Given the interplay between metabolic homeostasis and inflammation,^25^ we next examined the expression of genes including *ppara*, *pparg*, and *ucp2* in hepatic and colonic tissues of DIO mice. Those genes were upregulated in DIO mice treated with *C. massiliensis* (Figure 2m–r), suggesting a favorable shift in metabolic state in host mice.

Together, those results suggest that *C. massiliensis* alleviated obesity-associated metabolic dysfunction, possibly by coordinately regulating gut hormone signaling, inflammatory responses, and metabolic homeostasis.

### *C. massiliensis* significantly modulated the gut metabolome

**Figure 3.**
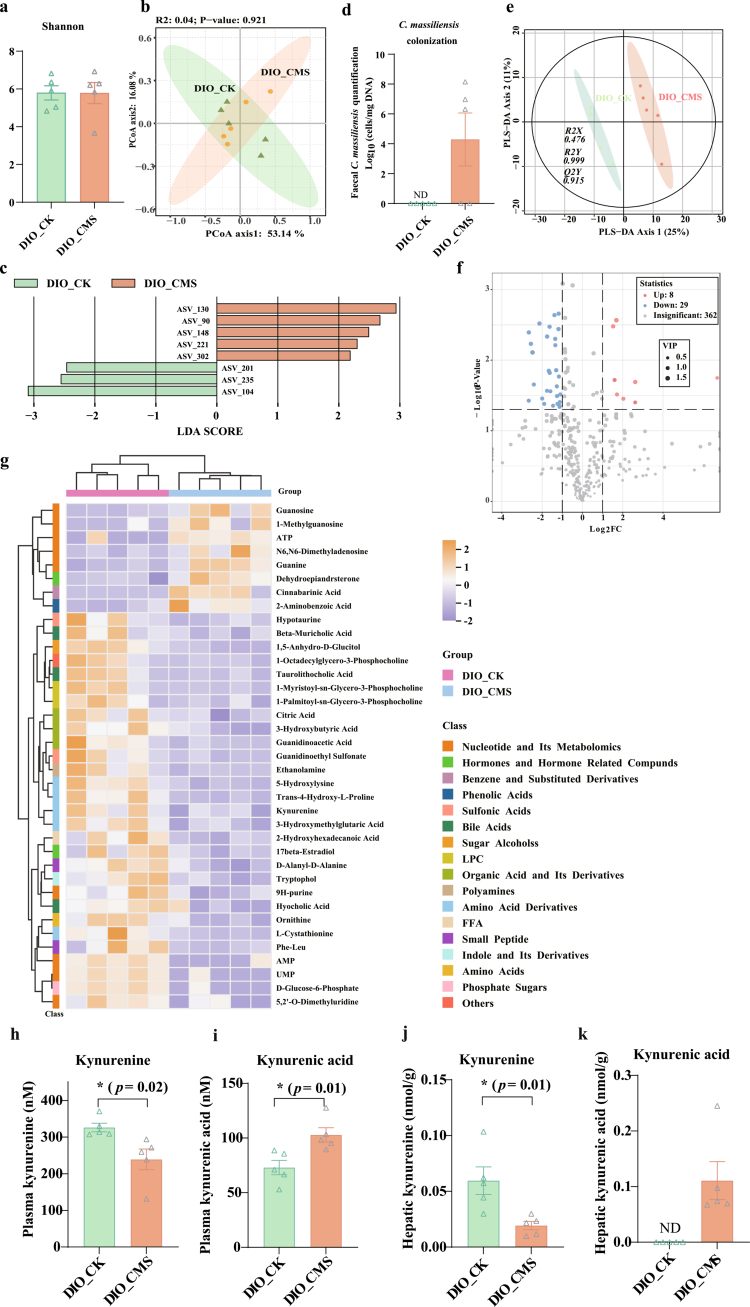
The effects of *C. massiliensis* on gut microbiota and metabolites in DIO mice. Shannon index (a), the principal coordinate analysis (PCoA) based on Bray–Curtis dissimilarity, with statistical significance assessed by PERMANOVA (b), the results of the LDA Effect Size (LEfSe) analysis, highlighting significant differences in taxonomic compositions at ASV levels (c), the qPCR-based absolute quantification of *C. massiliensis* copies in fecal sample of each group (d), the partial least squares discriminant analysis (PLS-DA) of DIO_CK and DIO_CMS group (*n* = 5 biologically independent samples for each) (e), the volcano plot (f), and the heatmap of 37 differential metabolites, identified based on a variable importance in projection (VIP) score > 1, *p*-value < 0.05, and a logarithmic |fold change (Log_2_ FC) > 1 (g), plasma kynurenine (h) and kynurenic acid level (i), hepatic kynurenine (j) and kynurenic acid level (k). The normality of the probability distribution was assessed using the Shapiro–Wilk test. For panels h–k, a two-tailed unpaired *t*-test was used for comparisons between two groups. Data are shown as mean ± SEM.

To further investigate the impact of *C. massiliensis* on host mice, we performed 16S rRNA amplicon sequencing and metabolic profiling on the cecal contents of mice. *C. massiliensis* administration had no significant effect on the α- or β-diversity of gut microbiota in DIO mice (Figure 3a and b). To further characterize potential taxonomic differences between groups, we conducted Linear Discriminant Analysis Effect Size (LEfSe) analysis at different taxonomic levels. At the genus and family levels, the LEfSe analysis did not reveal significant differences meeting the threshold (LDA > 2, *p* < 0.05, Tables S2 and S3). At the Amplicon Sequence Variant (ASV) level, 5 ASVs, including ASV_130 (*Lachnospiraceae*_NK4A136_group), ASV_90 (*Lachnospiraceae*), ASV_148 (Lachnoclostridium), ASV_221 (*UCG-009*), ASV_302 (*Oscillibacter*), were enriched in DIO mice treated with *C. massiliensis*, and 3 ASVs, including ASV_201 (*Clostridia*_vadinBB60_group), ASV_235 (*Candidatus*_*Saccharimonas*), ASV_104 (*Lachnospiraceae*), were enriched in the control group (Figure 3c). Subsequently, we quantified the *C. massiliensis* strain in fecal samples at the end of daily gavage using specific qPCR. The results showed that *C. massiliensis* has an abundance of approximately 2 × 10^7^ cells/mg of fecal metagenomic DNA in the gavaged mice, whereas no *C. massiliensis* was detected in the control group (Figure 3d). These results suggested that *C. massiliensis* administration did not significantly alter the overall gut microbiota composition. However, potential functional changes in the gut microbiota cannot be excluded, as we observed that 5 ASVs (ASV_130, ASV_90, ASV_148, ASV_221 (UCG-009), and ASV_302) were enriched in DIO mice treated with *C. massiliensis*, and the beneficial effects of *C. massiliensis* might partly result from the above bacterial taxa.

We then conducted targeted quantitative metabolomics on the cecal contents to assess the impact of *C. massiliensis* on gut metabolites. We identified a total of 399 metabolites belonging to 37 different chemical categories from all samples (Table S4). The distributions of these metabolites significantly discriminated between *C. massiliensis* treated group and the control, as shown by the Partial Least Squares Discriminant Analysis (PLS-DA, Figure 3e). Further differential analysis, incorporating *p*-values, fold changes, and Variable Importance in Projection (VIP) scores as described in the Methods section, identified 37 significantly altered metabolites from 17 categories. Among these, 8 metabolites were enriched and 29 were reduced in DIO mice treated with *C. massiliensis* (Figure 3f and g), with the most prominent changes observed in the “Nucleotide” class (*n* = 9). Specifically, levels of AMP, 9H-purine, guanine, guanosine, and N6, N6-dimethyladenosine were significantly decreased, while ATP levels were significantly elevated in the *C. massiliensis*-treated group compared to the control. Notably, several intermediates of the tryptophan metabolism pathway, including kynurenine, cinnabarinic acid, and tryptophol, were significantly altered following *C. massiliensis* treatment. Given the reported association of elevated kynurenine with metabolic dysfunction and obesity-related phenotypes,^44^ ^,^ ^45^ we further quantified kynurenine and kynurenic acid in plasma and liver. Consistent with the cecal metabolomic findings, *C. massiliensis* treatment significantly reduced kynurenine levels and increased kynurenic acid levels in both plasma and liver, compared with that of the control group (Figure 3h–k). These findings suggest that *C. massiliensis* modulated kynurenine metabolism, in addition to “nucleotide metabolism”, in mice.

### *C. massiliensis*-mediated metabolism of kynurenine contributed to the observed therapeutic effects

**Figure 4.**
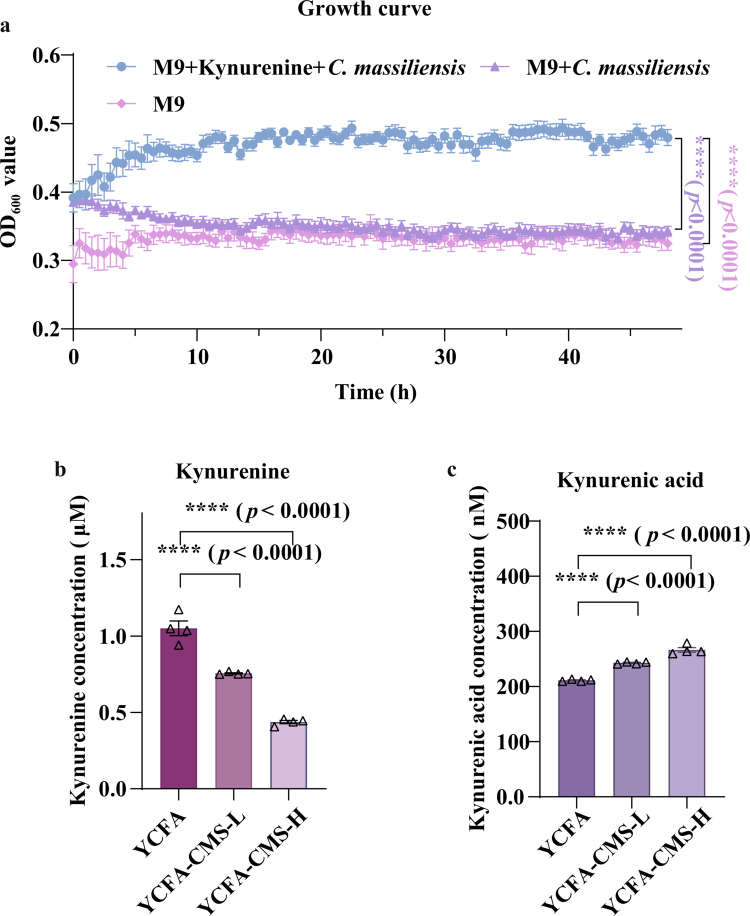
*In vitro* demonstration of kynurenine conversion by *C. massiliensis.* The growth curve of *C. massiliensis* (a) (*n* = 6 biologically independent samples for each), the concentration of kynurenine and kynurenic acid (b and c) (*n* = 4 biologically independent samples for each). The normality of the probability distribution was assessed using the Shapiro–Wilk test. For panels b–c, one-way ANOVA test was performed to analyze differences between YCFA and other groups. Data are shown as mean ± SEM.

Given the reduced kynurenine levels observed in cecal contents, plasma, and liver following *C. massiliensis* treatment, we next examined whether *C. massiliensis* could directly metabolize kynurenine. We cultured *C. massiliensis* in the presence and absence of kynurenine as the sole carbon source. Results show that *C. massiliensis* grew in the presence of kynurenine, suggesting that the strain can metabolize kynurenine (Figure 4a). Because cinnabarinic acid, a downstream product of kynurenine metabolism, was increased in the cecal metabolome, we further tested whether *C. massiliensis* could directly convert kynurenine into cinnabarinic acid. LC-MS analysis was performed in medium containing 1 μM kynurenine. However, cinnabarinic acid was not detected under either low (OD_600_ = 0.01, YCFA-CMS-L) or high (OD_600_ = 0.50, YCFA-CMS-H) inoculation conditions, suggesting that *C. massiliensis* alone did not convert kynurenine into cinnabarinic acid under these experimental settings.

To identify potential downstream products of kynurenine metabolism, we performed a combined analysis of the *C. massiliensis* genome and the KEGG database (Figure S1). The results revealed that *C. massiliensis* encodes arylformamidase (EC 3.5.1.9) and cysteine-S-conjugate beta-lyase (EC 4.4.1.13), suggesting its capacity to catalyze the conversion of L-formylkynurenine to kynurenine, and subsequently convert kynurenine into 4-(2-aminophenyl)-2,4-dioxobutanoate, an unstable intermediate rapidly cyclizes to form kynurenic acid.^46^ Consistently, LC-MS detected the characteristic peak of kynurenic acid in the culture supernatants, confirming that *C. massiliensis* is capable of converting kynurenine into kynurenic acid (Figure S2). To further validate this metabolic activity, we performed quantitative analysis of kynurenine consumption and kynurenic acid production. After 72 h of incubation at 37°C, the OD_600_ increased from 0.01 to 0.07 ± 0.008 in the YCFA-CMS-L group, and from 0.5 to 0.66 ± 0.02 in the YCFA-CMS-H group, indicating bacterial growth during incubation. Accordingly, the kynurenine conversion rates were 28% and 58.4% in the low- and high-inoculum groups, respectively. In parallel, kynurenic acid levels increased to 31.5 ± 1.2 nM and 55 ± 3.7 nM (Figure 4b and c). Previous studies have shown that elevated kynurenine promotes inflammation and metabolic dysfunction, whereas reducing kynurenine levels helps alleviate these abnormalities.^25^ ^,^ ^44^ ^,^ ^45^ ^,^ ^47^ We observed that *C. massiliensis*-treated mice reduced kynurenine levels, attenuated inflammation and improved metabolic phenotypes, supporting the beneficial effects of *C. massiliensis*.

### *C. massiliensis* alleviates kynurenine-induced hepatocellular lipid accumulation and metabolic dysregulation

**Figure 5.**
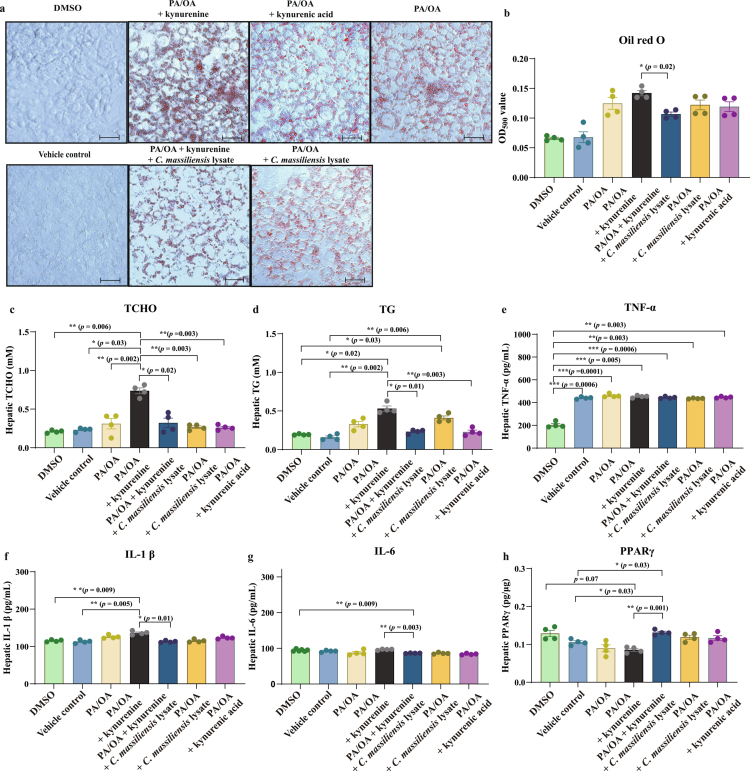
*C. massiliensis* lysate alleviates kynurenine-induced lipid accumulation, inflammation, and PPARγ suppression in AML12 hepatocytes. Representative Oil Red O staining images of AML12 cells treated (scale bar = 100 μm) (a), quantitative analysis of Oil Red O staining measured by absorbance at OD_500_ nm (b), TCHO level (c), TG level (d), TNF-α level (e), IL-1β level (f), IL-6 level (g), PPARγ level (h) in AML12 cells (*n* = 4 biologically independent samples for each). The normality of the probability distribution was assessed using the Shapiro–Wilk test. For panels b–g, group comparisons were performed using one-way ANOVA. For panel h, the Kruskal-Wallis test was applied to assess differences between groups. Data are shown as mean ± SEM.

Having shown that *C. massiliensis* reduced kynurenine levels *in vivo* and converted kynurenine into kynurenic acid *in vitro*, we next examined whether kynurenine directly impairs hepatocellular metabolic homeostasis and whether *C. massiliensis* could counteract this effect. A palmitic acid/oleic acid (PA/OA)-induced lipid accumulation model was established in murine hepatocyte AML12 cells. Cells were treated with DMSO, vehicle control, PA/OA, PA/OA + kynurenine, PA/OA + kynurenine + *C. massiliensis* lysate, PA/OA + *C. massiliensis* lysate, or PA/OA + kynurenic acid, respectively. After 24 h of treatment, Oil Red O staining was performed. Microscopic analysis showed negligible lipid accumulation in the DMSO and vehicle control groups, whereas abundant lipid droplets were observed after PA/OA treatment (Figure 5a). Oil Red O quantification showed that kynurenine further increased lipid accumulation under PA/OA stimulation, although without statistical significance. Notably, co-treatment with kynurenine and *C. massiliensis* lysate significantly reduced lipid deposition by approximately 24.9% compared with PA/OA plus kynurenine treatment (Figure 5b). In contrast, neither kynurenic acid nor *C. massiliensis* lysate alone exhibited a significant effect on lipid accumulation under PA/OA stimulation.

We further examined the effects of different treatments on intracellular TCHO, TG, and inflammatory cytokines, including TNF-α, IL-1β, IL-6. PA/OA treatment did not significantly alter TCHO or TG levels compared with DMSO and vehicle controls, whereas 100 μM kynurenine significantly increased both parameters. Supplementation with *C. massiliensis* lysate effectively reversed those changes, while neither kynurenic acid nor *C. massiliensis* lysate alone had significant effects (Figure 5c and d). Regarding inflammatory responses, kynurenine did not further increase TNF-α levels under PA/OA-treated conditions (Figure 5e). However, kynurenine significantly increased IL-1β secretion, which was significantly reduced by *C. massiliensis* lysate (Figure 5f). Kynurenine also tended to increase IL-6 levels, whereas *C. massiliensis* lysate significantly reduced IL-6 under kynurenine-treated conditions (Figure 5g). Those findings suggest that *C. massiliensis* lysate effectively counteracts kynurenine-induced metabolic dysregulation and inflammatory responses in hepatocytes.

Finally, we assessed the effects of different treatments on PPARγ levels. PA/OA treatment showed a decreasing trend in PPARγ expression without statistical significance, whereas kynurenine significantly suppressed PPARγ expression. This inhibitory effect was reversed by *C. massiliensis* lysate, while neither lysate alone nor kynurenic acid affected PPARγ expression (Figure 5h).

Collectively, those results show that *C. massiliensis* treatment attenuated kynurenine-induced lipid accumulation, inflammatory responses, and PPARγ suppression in hepatocytes. Together with the observed *in vivo* reduction of kynurenine and *in vitro* kynurenine-metabolizing activity of *C. massiliensis*, our findings provided evidence at cellular level that kynurenine metabolism of *C. massiliensis* is beneficial to host.

## Discussion

*Christensenellaceae* has long been considered inversely correlated with host BMI,^10‐13^ and some members of *Christensenellaceae*, such as *C. minuta* and *L. tenuis*, have been reported to exert weight-reducing efficacy.^14^ ^,^ ^16^ Although members of the *Christensenellaceae* family are generally low in abundance within the gut microbiota, they exhibit notable species diversity.^15^ Importantly, different strains may exert distinct effects on host health or disease. For instance, *Christensenella hongkongensis* has been previously reported to cause bacteremia in human hosts,^48^ ^,^ ^49^ indicating potential pathogenicity under certain conditions. In contrast, *L. tenuis* has been shown to alleviate host obesity and metabolic disorders by modulating gut hormone levels, including GLP-1 and PYY.^16^ *Christensenella tenuis* has been shown to alleviate endotoxemia and metabolic disorders in diet‑induced obese mice by producing free bile acids via bile salt hydrolase (BSH) activity; these free bile acids directly bind to intestinal lipopolysaccharide (LPS), form non‑membrane‑permeable complexes, and thereby block LPS translocation from the gut into the circulation, while also upregulating GLP-1 and TGR5 signaling to improve glucose and lipid metabolism.^50^ In this study, we transitioned from *in silico* analysis to experimental validation, demonstrating for the first time that *C. massiliensis* alleviates obesity and related metabolic disorders in DIO mice, highlighting its potential as a promising next-generation probiotic. The mechanism proposed for *C. massiliensis* is distinct from the previously reported ones of *Christensenellaceae* members.^14^ ^,^ ^16^ *C. minuta* improves host metabolism by producing 3-O-acyl bile acids that act as intestinal FXR antagonists,^14^ whereas *C. massiliensis* lacks the ability to synthesize these compounds. Our data revealed that *C. massiliensis* exerts metabolic benefits by modulating tryptophan-kynurenine metabolism. Besides, indirect beneficial effects to host mice cannot be excluded, as we observed that *C. massiliensis* administration triggered limited changes in gut microbiota composition. Taken all together, we propose that *Christensenellaceae* members regulate host metabolism through distinct and diverse pathways.

Based on our study, we propose that the beneficial effects on host mice are mainly attributed to the metabolism of *C. massiliensis*. Specific qPCR confirmed that the administered *C. massiliensis* strain was detectable in fecal samples during daily gavage. Short or transient colonization of gut microbes in host does not necessarily preclude their probiotic efficacy. For example, *Akkermansia muciniphila* shows baseline-dependent colonization efficiency and clinical response, and many orally administered probiotics colonize the gut only to a limited extent.^51‐53^ Importantly, *C. massiliensis* administration significantly altered metabolites in host mice, for example, significantly reduced cecal kynurenine levels and increased cecal ATP content. These intraluminal metabolic changes were accompanied by *in vitro* evidence that *C. massiliensis* metabolizes kynurenine and converts it into kynurenic acid. Previous studies have shown that kynurenine exacerbates obesity and metabolic disorders by promoting inflammation and disrupting metabolic homeostasis through pathways such as the AhR/STAT3/IL-6 signaling cascade.^25^ ^,^ ^54^ Similarly, DIO mice treated with *C. massiliensis* showed reduced kynurenine levels, which was accompanied by a decrease in inflammatory cytokines like IL-6 and an upregulation of genes associated with metabolic homeostasis. The hepatocyte experiments showed that kynurenine induced lipid accumulation, inflammatory responses, and PPARγ suppression, whereas *C. massiliensis* lysate reversed these effects. In addition, we found that the product of kynurenine metabolism by *C. massiliensis* is kynurenic acid. Previous studies have indicated that kynurenic acid acts as an agonist for signaling pathways such as GPR35, AMPK, and PPARγ, which are involved in fat metabolism, metabolic regulation, and inflammation suppression, thereby contributing to the alleviation of obesity and metabolic disorders in the host.^27^ ^,^ ^55^ ^,^ ^56^ Together, these findings support kynurenine degradation as a mechanism that may contribute to the beneficial metabolic effects of *C. massiliensis*.

In this study, we tried to disclose the genetics for kynurenine degradation and kynurenic acid production. The analysis of the *C. massiliensis* genome and KEGG database did not reveal the presence of genes encoding indoleamine 2,3-dioxygenase (IDO, EC:1.13.11.52) or tryptophan 2,3-dioxygenase (TDO, EC:1.13.11.11), suggesting that *C. massiliensis* is unlikely to directly catalyze the conversion of tryptophan to L-formylkynurenine. This step may instead be carried out by other members of the gut microbiota, highlighting the importance of microbial cross-feeding interactions in modulating tryptophan-kynurenine metabolism.^57^ In addition, we also observed a significant increase in cinnabarinic acid levels following *C. massiliensis* treatment. Previous studies have reported that cinnabarinic acid promotes lipolysis and thermogenic responses in adipose tissue, thereby enhancing glucose and lipid metabolism and controlling weight in DIO.^58^ However, LC-MS analysis indicated that *C. massiliensis* did not directly produce cinnabarinic acid under the tested conditions. Although cinnabarinic acid is not directly synthesized by *C. massiliensis* in this study, previous studies have reported that other gut microbes are capable of synthesizing cinnabarinic acid.^59^ ^,^ ^60^ Indeed, we observed the enrichments of several bacterial taxa associated with *C. massiliensis* treatment. Thus, we consider it very likely that the complete conversion of kynurenine and productions of kynurenic and cinnabarinic acids were results of gut microbial metabolic interactions rather than direct production by *C. massiliensis*.

We observed that *C. massiliensis* administration significantly reduced voluntary food intake in DIO mice. This was accompanied by increased plasma glucagon and GLP-1 levels, upregulation of ileal GLP-1 receptor (*glp1r*) expression, and decreased ghrelin levels, four markers known to influence host appetite and obesity.^61‐65^ These hormonal changes provide a plausible explanation for the reduced food intake observed in treated mice. The regulation of body weight, glucose homeostasis, and lipid metabolism indicates overall metabolic benefits.

This work has several limitations that should be addressed in future studies. First, the absence of metabolic cage analyses prevents us from distinguishing whether the reduced body weight and adiposity result from decreased energy intake, increased energy expenditure, or both; therefore, the observed metabolic benefits should be interpreted as integrated outcomes rather than direct evidence of altered energy expenditure. Second, a genetic manipulation system for *C. massiliensis* is currently unavailable, which precludes definitive causal validation of the kynurenine degradation pathway. Third, the long-term colonization stability and safety profile of *C. massiliensis* remain to be systematically evaluated, including its persistence beyond the treatment window and potential risks such as translocation or opportunistic infection. Fourth, bacterial lysate rather than live *C. massiliensis* was used in the in vitro hepatocyte experiments; while this enables controlled assessment, it may not fully recapitulate the effects of viable probiotics that continuously produce bioactive metabolites and interact dynamically with host cells.

From a translational perspective, several issues remain to be addressed before *C. massiliensis* or other *Christensenellaceae* members can be developed as a next-generation probiotic. Firstly, its long-term safety to human beings should be evaluated clinically. Secondly, because orally administered probiotics show limited colonization, future studies are needed to optimize dose frequency and probiotic formulation to improve probiotic viability, intestinal delivery and colonization. Thirdly, host-specific factors, including baseline gut microbiota composition and host genetic background, may influence strain persistence and treatment response, suggesting that host-strain compatibility should be considered. Finally, the ecological interactions between *C. massiliensis* and other gut microbes should be further explored to better define its role within the microbiome.

## Supplementary Material

Supplementary material Supplementary figures.docx

Supplementary tables.xlsx Supplementary tables.xlsx

## Data Availability

Data is deposited in National Microbiology Data Center (NMDC) with accession numbers NMDC10019725. (https://nmdc.cn/resource/genomics/project/detail/NMDC10019725).

## References

[1] Reddy KS , Yusuf S . Emerging epidemic of cardiovascular disease in developing countries. Circulation. 1998;97:596–601. doi: 10.1161/01.CIR.97.6.596. 9494031

[2] Lonardo A , Byrne CD , Caldwell SH , Cortez-Pinto H , Targher G . Global epidemiology of nonalcoholic fatty liver disease: meta-analytic assessment of prevalence, incidence, and outcomes. Hepatology. 2016;64:1388–1389. doi: 10.1002/hep.28584. 27038241

[3] Lakka HM , Laaksonen D E , Lakka TA , Niskanen LK , Kumpusalo E , Tuomilehto J , Salonen JT . The metabolic syndrome and total and cardiovascular disease mortality in middle-aged men. Jama-J Am Med Assoc. 2002;288:2709–2716. doi: 10.1001/jama.288.21.2709 ​​​​​​. 12460094

[4] Jaacks LM , Vandevijvere S , Pan A , McGowan CJ , Wallace C , Imamura F , Mozaffarian D , Swinburn B , Ezzati M . The obesity transition: stages of the global epidemic. Lancet Diabetes Endo. 2019;7:231–240. doi: 10.1016/S2213-8587(19)30026-9. PMC7360432 30704950

[5] Zheng Y , Ley SH , Hu FB . Global aetiology and epidemiology of type 2 diabetes mellitus and its complications. Nat Rev Endocrinol. 2018;14:88–98. doi: 10.1038/nrendo.2017.151. 29219149

[6] Finelli C , Padula MC , Martelli G , Tarantino G . Could the improvement of obesity-related co-morbidities depend on modified gut hormones secretion?. World J Gastroenterol. 2014;20:16649–16664. doi: 10.3748/wjg.v20.i44.16649. 25469034 PMC4248209

[7] Chakaroun RM , Massier L , Heintz-Buschart A , Said N , Fallmann J , Crane A , Schütz T , Dietrich A , Blüher M , Stumvoll M , et al. Circulating bacterial signature is linked to metabolic disease and shifts with metabolic alleviation after bariatric surgery. Genome Med. 2021;13:105. doi: 10.1186/s13073-021-00919-6.​​​​​​ 34158092 PMC8218394

[8] Fan Y , Pedersen O . Gut microbiota in human metabolic health and disease. Nat Rev Microbiol. 2021;19:55–71. doi: 10.1038/s41579-020-0433-9. 32887946

[9] Sergeev IN , Aljutaily T , Walton G , Huarte E . Effects of synbiotic supplement on human gut microbiota, body composition and weight loss in obesity. Nutrients. 2020;12. doi: 10.3390/nu12010222. PMC7019807 31952249

[10] Goodrich JK , Waters JL , Poole AC , Sutter JL , Koren O , Blekhman R , Beaumont M , Van Treuren W , Knight R , Bell JT , et al. Human genetics shape the gut microbiome. Cell. 2014;159:789–799. doi: 10.1016/j.cell.2014.09.053. 25417156 PMC4255478

[11] Li X , He Y , Zhou H , Zeng N . Regional distribution of *Christensenellaceae* and its associations with metabolic syndrome based on a population-level analysis. PeerJ. 2020;8:e9591. doi:10.7717/peerj.9591. 32832265 PMC7413085

[12] Waters JL , Ley RE . The human gut bacteria *Christensenellaceae* are widespread. BMC Biol. 2019;17:83. doi: 10.1186/s12915-019-0699-4. 31660948 PMC6819567

[13] Alemán JO , Bokulich NA , Swann JR , Walker JM , De Rosa JC , Battaglia T , Costabile A , Pechlivanis A , Liang Y , Breslow JL , et al. Fecal microbiota and bile acid interactions with systemic and adipose tissue metabolism in diet-induced weight loss of obese postmenopausal women. J Transl Med. 2018;16:244 244. doi: 10.1186/s12967-018-1619-z. 30176893 PMC6122649

[14] Liu C , Du M , Xie L , Wang W , Chen B , Yun C , Sun X , Luo X , Jiang Y , Qiao S , et al. Gut commensal *Christensenella minuta* modulates host metabolism via acylated secondary bile acids. Nat Microbiol. 2024;9:434–450. doi: 10.1038/s41564-023-01570-0. 38233647

[15] Sun XW , Huang H , Wang X , Wei R , Niu H , Chen H , Luo M , Abdugheni R , Liu F , Jiang H . Christensenella strain resources, genomic/metabolomic profiling, and association with host at species level. Gut Microbes. 2024;16:2347725. doi: 10.1080/19490976.2024.2347725. 38722028 PMC11085954

[16] Jiang Y , Du M , Xie L , Zhang Y , Bi M , Liu C . The human-derived novel gut commensal *Luoshenia tenuis* regulates body weight and food intake in mice. Food Sci Hum Well. 2024;13:830–841. doi: 10.26599/Fshw.2022.9250071.

[17] Huffman KM , Shah SH , Stevens RD , Bain JR , Muehlbauer M , Slentz CA , Tanner CJ , Kuchibhatla M , Houmard JA , Newgard CB , et al. Relationships between circulating metabolic intermediates and insulin action in overweight to obese, inactive men and women. Diabetes Care. 2009;32:1678–1683. doi: 10.2337/dc08-2075. 19502541 PMC2732163

[18] Fiehn O , Garvey WT , Newman JW , Lok KH , Hoppel CL , Adams SH , Gimble JM . Plasma metabolomic profiles reflective of glucose homeostasis in non-diabetic and type 2 diabetic obese African-American women. PLoS One. 2010;5:e15234. doi: 10.1371/journal.pone.0015234. 21170321 PMC3000813

[19] Menge BA , Schrader H , Ritter PR , Ellrichmann M , Uhl W , Schmidt WE , Meier JJ . Selective amino acid deficiency in patients with impaired glucose tolerance and type 2 diabetes. Regul Peptides. 2010;160:75–80. doi: 10.1016/j.regpep.2009.08.001. 19695292

[20] Tai ES , Tan MLS , Stevens RD , Low YL , Muehlbauer MJ , Goh DLM , Ilkayeva OR , Wenner BR , Bain JR , Lee JJM , et al. Insulin resistance is associated with a metabolic profile of altered protein metabolism in Chinese and Asian-Indian men. Diabetologia. 2010;53:757–767. doi: 10.1007/s00125-009-1637-8. 20076942 PMC3753085

[21] Adibi SA . Influence of dietary deprivations on plasma concentration of free amino acids of man. J Appl Physiol. 1968;25:52–57. doi: 10.1152/jappl.1968.25.1.52. ​​​​​​ 5661154

[22] Felig P , Marliss E , Cahill GF . Plasma amino acid levels and insulin secretion in obesity. New Engl J Med. 1969;281:811–816. doi: 10.1056/Nejm196910092811503. 5809519

[23] Zeng MM , Liang Y , Li H , Wang M , Chen X , Zhou N , Cao D , Wu J . Plasma metabolic fingerprinting of childhood obesity by GC/MS in conjunction with multivariate statistical analysis. J Pharmaceut Biomed. 2010;52:265–272. doi: 10.1016/j.jpba.2010.01.002. 20092977

[24] Rojas IY , Moyer BJ , Ringelberg CS , Wilkins OM , Pooler DB , Ness DB , Coker S , Tosteson TD , Lewis LD , Chamberlin MD , et al. Kynurenine-induced aryl hydrocarbon receptor signaling in mice causes body mass gain, liver steatosis, and hyperglycemia. Obesity. 2021;29:337–349. doi: 10.1002/oby.23065. 33491319 PMC10782555

[25] Huang T , Song J , Gao J , Cheng J , Xie H , Zhang L , Wang Y , He J , Liu S , Yu Q , et al. Adipocyte-derived kynurenine promotes obesity and insulin resistance by activating the AhR/STAT3/IL-6 signaling. Nat Commun. 2022;13:3489. doi: 10.1038/s41467-022-31126-5. 35715443 PMC9205899

[26] Dadvar S , Ferreira DMS , Cervenka I , Ruas JL . The weight of nutrients: kynurenine metabolites in obesity and exercise. J Intern Med. 2018;284:519–533. doi: 10.1111/joim.12830. 30141532

[27] Agudelo LZ , Ferreira DM , Cervenka I , Bryzgalova G , Dadvar S , Jannig PR , Pettersson-Klein AT , Lakshmikanth T , Sustarsic EG , Porsmyr-Palmertz M , et al. Kynurenic acid and Gpr35 regulate adipose tissue energy homeostasis and inflammation. Cell Metab. 2018;27:378–392.e375. doi: 10.1016/j.cmet.2018.01.004. 29414686

[28] Oxenkrug G . Insulin resistance and dysregulation of tryptophan-kynurenine and kynurenine-nicotinamide adenine dinucleotide metabolic pathways. Mol Neurobiol. 2013;48:294–301. doi: 10.1007/s12035-013-8497-4. 23813101 PMC3779535

[29] Qiao S , Liu C , Sun L , Wang T , Dai H , Bao L , Li H . Gut *Parabacteroides merdae* protects against cardiovascular damage by enhancing branched-chain amino acid catabolism. Nat Metab. 2022;4:1271–1286. doi: 10.1038/s42255-022-00649-y. 36253620

[30] Wu HZ , Ballantyne CM . Metabolic inflammation and insulin resistance in obesity. Circ Res. 2020;126:1549–1564. doi: 10.1161/Circresaha.119.315896. 32437299 PMC7250139

[31] Rohm TV , Meier DT , Olefsky JM , Donath MY . Inflammation in obesity, diabetes, and related disorders. Immunity. 2022;55:31–55. doi: 10.1016/j.immuni.2021.12.013. 35021057 PMC8773457

[32] Abouelela ME , Helmy YA . Next-generation probiotics as novel therapeutics for improving human health: current trends and future perspectives. Microorganisms. 2024;12. doi: 10.3390/microorganisms12030430. PMC10972033 38543481

[33] Jan TWF , Negi R , Sharma B , Kumar S , Singh S , Rai AK , Shreaz S , Rustagi S , Chaudhary N , Kaur T , et al. Next generation probiotics for human health: an emerging perspective. Heliyon. 2024;10:e35980. doi: 10.1016/j.heliyon.2024.e35980. 39229543 PMC11369468

[34] Al-Fakhrany OM , Elekhnawy E . Next-generation probiotics: the upcoming biotherapeutics. Mol Biol Rep. 2024;51:505. doi: 10.1007/s11033-024-09398-5. 38619680 PMC11018693

[35] Liu C , Du M , Abuduaini R , Yu H , Li D , Wang Y , Zhou N , Jiang M , Niu P , Han S , et al. Enlightening the taxonomy darkness of human gut microbiomes with a cultured biobank. Microbiome. 2021;9:119. doi: 10.1186/s40168-021-01064-3. 34020714 PMC8140505

[36] Virtue S , Vidal-Puig A . GTTs and ITTs in mice: simple tests, complex answers. Nat Metab. 2021;3:883–886. doi: 10.1038/s42255-021-00414-7. 34117483

[37] Livak KJ , Schmittgen TD . Analysis of relative gene expression data using real-time quantitative PCR and the 2(-Delta Delta C(T)) Method. Methods. 2001;25:402–408. doi: 10.1006/meth.2001.1262. 11846609

[38] Qiao S , Bao L , Wang K , Sun S , Liao M , Liu C , Zhou N , Ma K , Zhang Y , Chen Y . Activation of a specific gut Bacteroides–folate–liver axis benefits for the alleviation of nonalcoholic hepatic steatosis. Cell Rep. 2020;32:108005. doi: 10.1016/j.celrep.2020.108005. 32783933

[39] Fujii H , Ikura Y , Arimoto J , Sugioka K , Iezzoni JC , Park SH , Naruko T , Itabe H , Kawada N , Caldwell SH , et al. Expression of perilipin and adipophilin in nonalcoholic fatty liver disease; relevance to oxidative injury and hepatocyte ballooning. J Atheroscler Thromb. 2009;16:893–901. doi: 10.5551/jat.2055. 20032580

[40] Schirra J , Nicolaus M , Roggel R , Katschinski M , Storr M , Woerle HJ , Göke B . Endogenous glucagon-like peptide 1 controls endocrine pancreatic secretion and antro-pyloro-duodenal motility in humans. Gut. 2006;55:243–251. doi: 10.1136/gut.2004.059741. 15985560 PMC1856508

[41] Han H , Yi B , Zhong R , Wang M , Zhang S , Ma J , Yin Y , Chen L . From gut microbiota to host appetite: gut microbiota-derived metabolites as key regulators. Microbiome. 2021;9:162. doi: 10.1186/s40168-021-01093-y. 34284827 PMC8293578

[42] Xu H . Obesity and metabolic inflammation. Drug Discov Today Dis Mech. 2013;10:e21–e25. doi: 10.1016/j.ddmec.2013.03.006. PMC3758492 24003334

[43] Ellulu MS , Patimah I , Khaza'ai H , Rahmat A , Abed Y . Obesity and inflammation: the linking mechanism and the complications. Arch Med Sci. 2017;13:851–863. doi: 10.5114/aoms.2016.58928. 28721154 PMC5507106

[44] Sas K , Szabó E , Vécsei L . Mitochondria, oxidative stress and the kynurenine system, with a focus on ageing and neuroprotection. Molecules. 2018;23:191. doi: 10.3390/molecules23010191. 29342113 PMC6017505

[45] Mor A , Tankiewicz-Kwedlo A , Krupa A , Pawlak D . Role of kynurenine pathway in oxidative stress during neurodegenerative disorders. Cells. 2021;10:1603. doi: 10.3390/cells10071603. 34206739 PMC8306609

[46] Koper K , Han SW , Pastor DC , Yoshikuni Y , Maeda HA . Evolutionary origin and functional diversification of aminotransferases. J Biol Chem. 2022;298:102122. doi: 10.1016/j.jbc.2022.102122. 35697072 PMC9309667

[47] Haq S , Grondin JA , Khan WI . Tryptophan-derived serotonin-kynurenine balance in immune activation and intestinal inflammation. FASEB J. 2021;35:e21888. doi: 10.1096/fj.202100702R. 34473368 PMC9292703

[48] Kamau E , Maliksi E , Kwan N , Garner OB , Yang S . Catabacter hongkongensis bacteremia identified by direct metagenomic sequencing of positive blood culture fluid, first case report in the US. Anaerobe. 2021;71:102421. doi: 10.1016/j.anaerobe.2021.102421. 34314867

[49] Itoh N , Akazawa N , Ishibana Y , Murakami H . Peritonitis with bacteremia due to *Christensenella hongkongensis* identified via ribosomal RNA sequencing in a Japanese patient with advanced colorectal adenocarcinoma: a case report. IDCases. 2023;32:e01797. doi: 10.1016/j.idcr.2023.e01797. 37214185 PMC10196759

[50] Jiang Y , Zhu J , Du M , Zhao Q , Huang H , Sun X , Wang L , Liu C . *Christensenella tenuis* alleviates endotoxemia and metabolic disorders via inhibition of intestinal lipopolysaccharide translocation. Sci China Life Sci. 2025;68:3711–3727. doi: 10.1007/s11427-025-3014-6. 40947463

[51] Zhang Y , Liu R , Chen Y , Cao Z , Bao R , Wang Y , Huang S , Pan S , Qin L , Ning G . *Akkermansia muciniphila* supplementation in patients with overweight/obese type 2 diabetes: efficacy depends on its baseline levels in the gut. Cell Metab. 2025;37:592–605.e596. doi: 10.1016/j.cmet.2024.12.010. 39879980

[52] Rasmussen TS , Mentzel CMJ , Danielsen MR , Jakobsen RR , Zachariassen LSF , Castro Mejia JL , Brunse A , Hansen LH , Nielsen DS . Fecal virome transfer improves proliferation of commensal gut *Akkermansia muciniphila* and unexpectedly enhances the fertility rate in laboratory mice. Gut Microbes. 2023;15:2208504. doi: 10.1080/19490976.2023.2208504. 37150906 PMC10167882

[53] Maldonado-Gómez MX , Martínez I , Bottacini F , O’Callaghan A , Ventura M , van Sinderen D , Hillmann B , Vangay P , Knights D , Hutkins RW , et al. Stable engraftment of AH1206 in the human gut depends on individualized features of the resident microbiome. Cell Host Microbe. 2016;20:515–526. doi: 10.1016/j.chom.2016.09.001. 27693307

[54] Mor A , Tankiewicz-Kwedlo A , Ciwun M , Lewkowicz J , Pawlak D . Kynurenines as a novel target for the treatment of inflammatory disorders. Cells. 2024;13:1259. doi: 10.3390/cells13151259. 39120289 PMC11311768

[55] Zhen D , Liu J , Zhang XD , Song Z . Kynurenic acid acts as a signaling molecule regulating energy expenditure and is closely associated with metabolic diseases. Front Endocrinol (Lausanne). 2022;13:847611. doi: 10.3389/fendo.2022.847611. 35282457 PMC8908966

[56] Liu JJ , Raynal S , Bailbé D , Gausseres B , Carbonne C , Autier V , Movassat J , Kergoat M , Portha B . Expression of the kynurenine pathway enzymes in the pancreatic islet cells. Activation by cytokines and glucolipotoxicity. Biochim Biophys Acta. 2015;1852:980–991. doi: 10.1016/j.bbadis.2015.02.001. 25675848

[57] Gao K , Mu CL , Farzi A , Zhu WY . Tryptophan metabolism: a link between the gut microbiota and brain. Advances in Nutrition (Bethesda, Md.). 2020;11:709–723. doi: 10.1093/advances/nmz127. 31825083 PMC7231603

[58] Hu SS , Luo L , Zhang H , Zhao S , Liu Z , Zeng L . Pu-erh tea increases the metabolite cinnabarinic acid to improve circadian rhythm disorder-induced obesity. Food Chem. 2022;394:133500. doi: 10.1016/j.foodchem.2022.133500. 35749873

[59] Dehhaghi M , Kazemi Shariat Panahi H , Guillemin GJ . Microorganisms, tryptophan metabolism, and kynurenine pathway: a complex interconnected loop influencing human health status. International journal of tryptophan research: IJTR. 2019;12:1178646919852996. doi: 10.1177/1178646919852996. 31258331 PMC6585246

[60] Anesi A , Berding K , Clarke G , Stanton C , Cryan JF , Caplice N , Ross RP , Doolan A , Vrhovsek U , Mattivi F . Metabolomic workflow for the accurate and high-throughput exploration of the pathways of tryptophan, tyrosine, phenylalanine, and branched-chain amino acids in human biofluids. J Proteome Res. 2022;21:1262–1275. doi: 10.1021/acs.jproteome.1c00946. 35380444 PMC9087329

[61] Habegger KM , Heppner KM , Geary N , Bartness TJ , DiMarchi R , Tschöp MH . The metabolic actions of glucagon revisited. Nat Rev Endocrinol. 2010;6:689–697. doi: 10.1038/nrendo.2010.187. 20957001 PMC3563428

[62] Kirsz K , Zieba DA . Ghrelin-mediated appetite regulation in the central nervous system. Peptides. 2011;32:2256–2264. doi: 10.1016/j.peptides.2011.04.010. 21524673

[63] Obradovic M , Sudar-Milovanovic E , Soskic S , Essack M , Arya S , Stewart AJ , Gojobori T , Isenovic ER . Leptin and obesity: role and clinical implication. Front Endocrinol. 2021;12 585887. doi: 10.3389/fendo.2021.585887. PMC8167040 34084149

[64] Perry RJ , Zhang D , Guerra MT , Brill AL , Goedeke L , Nasiri AR , Rabin-Court A , Wang Y , Peng L , Dufour S , et al. Glucagon stimulates gluconeogenesis by INSP3R1-mediated hepatic lipolysis. Natur. 2020;579:279–283. doi: 10.1038/s41586-020-2074-6. PMC7101062 32132708

[65] Drucker DJ . Mechanisms of action and therapeutic application of glucagon-like Peptide-1. Cell Metab. 2018;27:740–756. doi: 10.1016/j.cmet.2018.03.001. 29617641

