# Supplementary material for: Christensenella massiliensis reduces kynurenine levels and alleviates obesity and related metabolic disorders in model mice
Source: Gut Microbes. 2026 Jul 11;18(1):2701382. doi: 10.1080/19490976.2026.2701382 (PMC13360501; doi:10.1080/19490976.2026.2701382)
Supplement: Supplementary material — Supplementary figures.docx [file KGMI_A_2701382_SM2585.docx]

***Christensenella massiliensis* reduces kynurenine levels and alleviates obesity and related metabolic disorders in model mice**

Meng-Xuan Du^1^, Wenzhao Wang^2^, Min-Zhi Jiang^1^, Xin-Wei Sun^1^, Lei Sun^1, 3^, Chang Liu^1*^, Shuang-Jiang Liu^1,2*^

Mengxuan Du: ^1^State Key Laboratory of Microbial Technology, Shandong University, Qingdao 266000, China. dumx@sdu.edu.cn

Wenzhao Wang: ^2^State Key Laboratory of Microbial Diversity and Innovative Application, Institute of Microbiology, Chinese Academy of Sciences, Beijing 100101, China. [wangwz@im.ac.cn](mailto:wangwz@im.ac.cn)

Min-Zhi Jiang: ^1^State Key Laboratory of Microbial Technology, Shandong University, Qingdao 266000, China. [jiangminzhi2006@126.com](mailto:jiangminzhi2006@126.com)

Xinwei Sun: ^1^State Key Laboratory of Microbial Technology, Shandong University, Qingdao 266000, China. [xinwei98s@sina.com](mailto:xinwei98s@sina.com)

Lei Sun: ^1^State Key Laboratory of Microbial Technology, Shandong University, Qingdao 266000, China.

^3^Shandong Provincial Key Laboratory of Animal Cell and Developmental Biology, School of Life Sciences, Shandong University, Qingdao 266000, China. [sunlei0227@sdu.edu.cn](mailto:sunlei0227@sdu.edu.cn)

Chang Liu: ^1^State Key Laboratory of Microbial Technology, Shandong University, Qingdao 266000, China. [liu.c@sdu.edu.cn](mailto:liu.c@sdu.edu.cn)

Shuang-Jiang Liu: ^1^State Key Laboratory of Microbial Technology, Shandong University, Qingdao 266000, China; ^2^State Key Laboratory of Microbial Diversity and Innovative Application, Institute of Microbiology, Chinese Academy of Sciences, Beijing 100101, China. [liusj@sdu.edu.cn](mailto:liusj@sdu.edu.cn)

*Corresponding authors: Chang Liu ([liu.c@sdu.edu.cn](mailto:liu.c@sdu.edu.cn)) and Shuang-Jiang Liu ([liusj@sdu.edu.cn](mailto:liusj@sdu.edu.cn)).


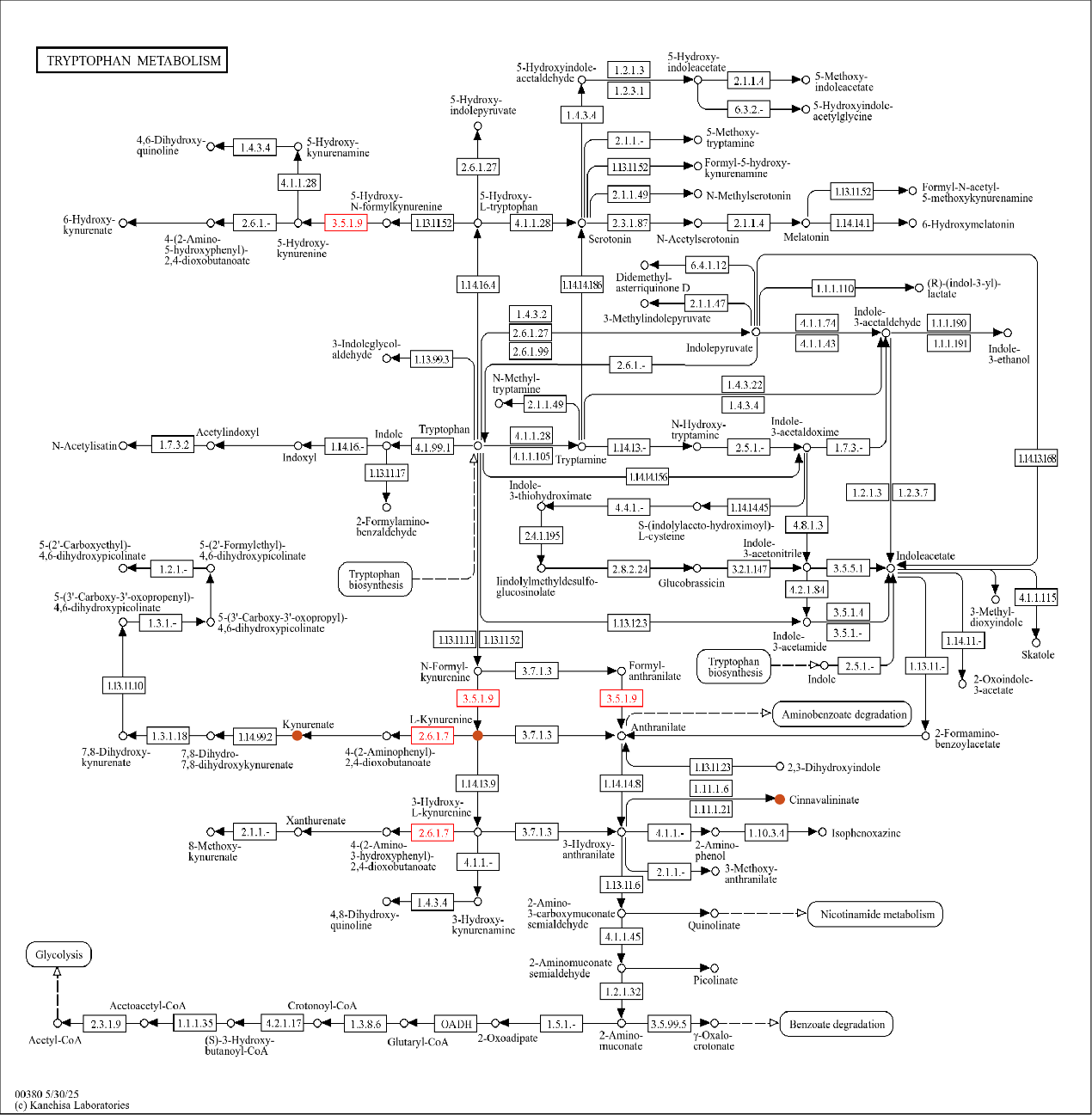


Fig.S1 Results of combined analysis of the C. massiliensis genome and KEGG pathway analysis. The enzymes encoded by the CMS genome were marked in red.


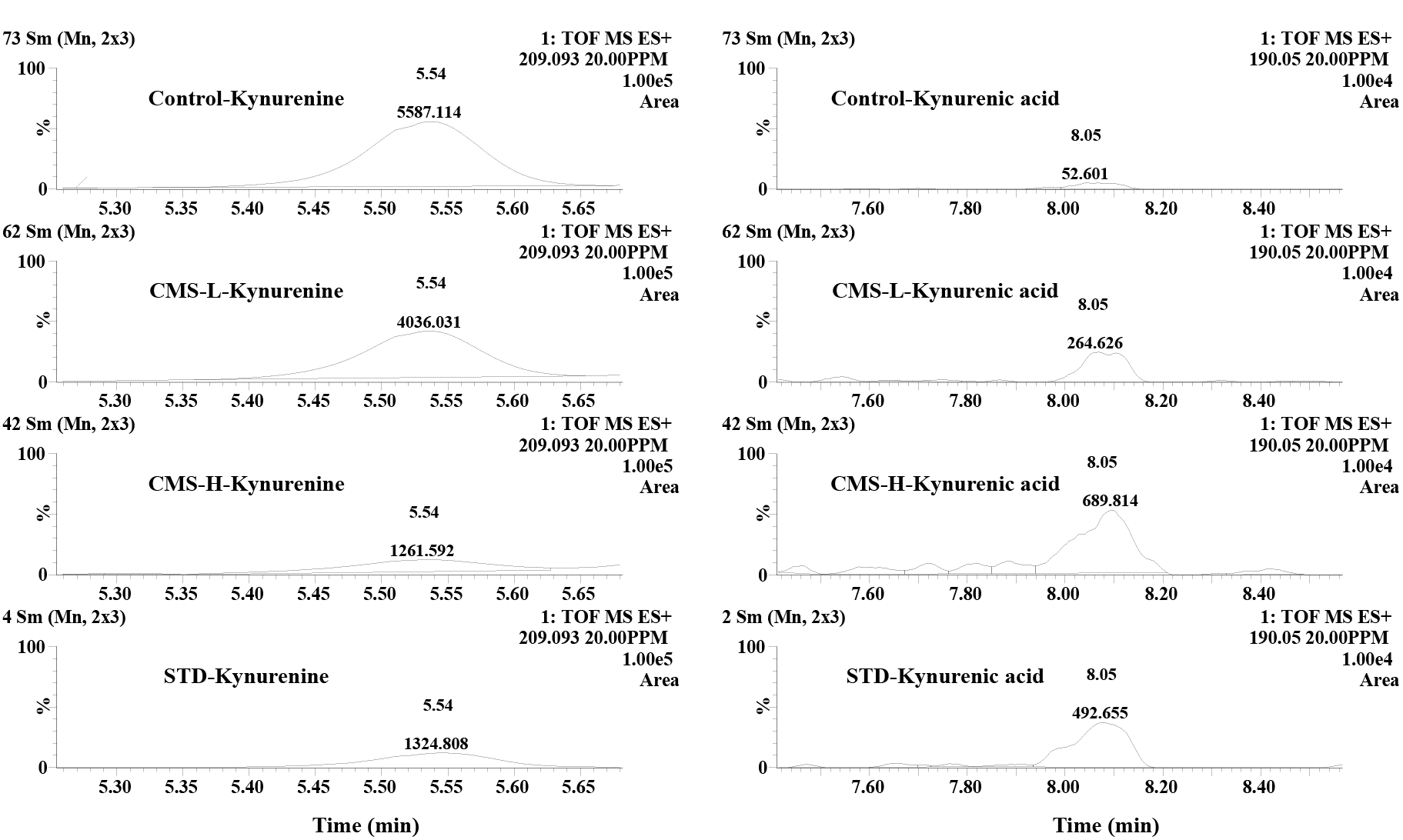


Fig. S2 Representative LC‑MS chromatograms of *Christensenella massiliensis* cultures for kynurenine metabolism.
